# Recording plasticity in neuronal activity in the rodent intrinsic cardiac nervous system using calcium imaging techniques

**DOI:** 10.3389/fnsyn.2023.1104736

**Published:** 2023-04-04

**Authors:** Joscelin E. G. Smith, Jesse L. Ashton, Liam P. Argent, Juliette E. Cheyne, Johanna M. Montgomery

**Affiliations:** ^1^Department of Physiology, University of Auckland, Auckland, New Zealand; ^2^Pūtahi Manawa, Centre for Heart Research, Auckland, New Zealand

**Keywords:** calcium imaging, ganglionated plexi, intrinsic cardiac nervous system, nicotinic acetylcholine receptors, atrial fibrillation, plasticity, neurons

## Abstract

The intrinsic cardiac nervous system (ICNS) is composed of interconnected clusters of neurons called ganglionated plexi (GP) which play a major role in controlling heart rate and rhythm. The function of these neurons is particularly important due to their involvement in cardiac arrhythmias such as atrial fibrillation (AF), and previous work has shown that plasticity in GP neural networks could underpin aberrant activity patterns that drive AF. As research in this field increases, developing new techniques to visualize the complex interactions and plasticity in this GP network is essential. In this study we have developed a calcium imaging method enabling the simultaneous recording of plasticity in neuronal activity from multiple neurons in intact atrial GP networks. Calcium imaging was performed with Cal-520 AM labeling in aged spontaneously hypertensive rats (SHRs), which display both spontaneous and induced AF, and age-matched Wistar Kyoto (WKY) controls to determine the relationship between chronic hypertension, arrhythmia and GP calcium dynamics. Our data show that SHR GPs have significantly larger calcium responses to cholinergic stimulation compared to WKY controls, as determined by both higher amplitude and longer duration calcium responses. Responses were significantly but not fully blocked by hexamethonium, indicating multiple cholinergic receptor subtypes are involved in the calcium response. Given that SHRs are susceptible to cardiac arrhythmias, our data provide evidence for a potential link between arrhythmia and plasticity in calcium dynamics that occur not only in cardiomyocytes but also in the GP neurons of the heart.

## Introduction

Cardiac ganglionated plexi (GP) are clusters of neurons located on the surface of the heart that play a major role in regulating heart rate and rhythm (Campos et al., 2018). The complex of multiple interconnected GPs together form the intrinsic cardiac nervous system (ICNS) that integrates afferent and efferent inputs from the central and peripheral nervous systems (Fedele and Brand, 2020). The GP play an important role in triggering arrhythmias such as atrial fibrillation (AF). For example, electrical stimulation of GPs induces AF in dogs (Scherlag et al., 2005) and humans (Lim et al., 2011; Salavatian et al., 2016), whereas the destruction of GPs can lower arrhythmia recurrence in AF patients (Katritsis et al., 2013). The precise role of GP in arrhythmias and AF, however, is not fully understood.

Within the GP, acetylcholine (ACh) is the primary neurotransmitter which binds to its corresponding nicotinic acetylcholine receptors (nAChRs) or muscarinic acetylcholine receptors (mAChRs) at presynaptic and postsynaptic terminals (Cuevas and Berg, 1998; Beker et al., 2003; Gotti et al., 2009; Brown, 2010). nAChRs and mAChRs have important functions in regulating neurotransmitter release, neuronal excitability, and synaptic plasticity (Gotti et al., 2009). nAChRs are inwardly rectifying ligand-gated ion channels permeable to Na^+^, K^+^, and Ca^2+^ (Del Signore et al., 2004) formed by complexes of α and β subunits. The three most common types of receptors are heteromeric α4β2 receptors, heteromeric α3β4 receptors and homomeric α7 receptors, all of which have been identified in the ICNS (Poth et al., 1997; Skok et al., 1999). nAChRs have an essential role in regulating heart rate as the application of nAChR antagonists has been shown to result in a loss of sinus rhythm including alteration of sinus cycle length and asystole (Bibevski et al., 2000; Deck et al., 2005), but the mechanism underlying this control is not fully understood. Direct injection of ACh into the epicardial fat pads that contain GPs in dogs provokes spontaneous or easily induced episodes of AF (Po et al., 2006), indicating a link between ACh receptors in regulating heart rate and rhythm in AF.

We have previously reported that the expression of specific nAChRs differs in the GP neurons of spontaneously hypertensive rats (SHRs) compared to Wistar Kyoto (WKY) controls, with SHRs showing a higher synaptic proportion of the highly calcium permeable α7 subunit containing AChRs (Ashton et al., 2020). Together with whole cell patch clamp electrophysiology observations of higher spontaneous synaptic activity in SHRs relative to WKYs, these data show chronic remodeling of GP cardiac neuron structure and function. As SHRs are particularly susceptible to cardiac arrhythmias such as AF and develop spontaneous AF with age (Scridon et al., 2012), this work suggests that changes in AChR signaling may contribute to the substrate for atrial arrhythmia in hypertensive heart disease in SHRs, but further research is needed to confirm their role.

Thus far, non-clinical GP research has centered on characterizing the electrical properties of individual GP neurons (Cuevas et al., 1997; Rimmer and Harper, 2006; Harper and Adams, 2021) or protein expression of fixed GP tissue (Richardson et al., 2003; Slavíkov et al., 2003; Hoover et al., 2009). These techniques have shown that each GP contains a heterogeneous population of neurons with distinct electrical, structural and molecular compositions (Selyanko, 1992; Edwards et al., 1995; Klemm et al., 1997), highlighting the system’s complexity. Specifically, cellular physiological recordings from GP neurons in whole atria or working heart-brainstem preparations have been performed using intracellular recordings and whole cell patch clamp techniques, meaning many questions regarding the network properties of the ICNS remain unanswered. The membranous connective tissue protecting the GP, as well as glial cells surrounding the individual neurons has provided a significant barrier for physiological access to GPs and this has hindered progress in the development of calcium imaging techniques in these cells.

In this study, we sought to develop a technique to simultaneously record the neurons in an intact functioning GP to give insight into the network-wide interactions of the different neuron types. As the Ca^2+^ dynamics of a neuron are indicative of their electrical activity, we aimed to develop a method to image calcium dynamics in the GP to provide us with a new perspective on ganglionic transmission and population activity patterns occurring within GP. Our data reveal the successful development of this method in intact GP, and show enhanced Ca^2+^ responses to postsynaptic ACh receptor activation in aged SHRs compared to age-matched WKY controls, providing a live read out of increased GP network excitability with chronic hypertension and arrhythmia.

## Methods

### Animals and atrial GP preparation

Male SHRs and WKY controls were imported from Charles River and aged from 16 to 20 months (300–500 g). All animal experiments were approved by the University of Auckland Animal Ethics Committee (approval number 2090) and conducted in accordance with the New Zealand Animal Welfare Act 1999.

Preparations containing multiple atrial GP were prepared as described previously (Ashton et al., 2020). Briefly, rats were deeply anaesthetized (5% isoflurane in O_2_, 4 min) and euthanized by cervical dislocation. A thoracotomy was performed and heparin (500 IU) was injected into the left ventricular cavity before cannulation and perfusion with 4^°^C carbogenated (5 CO_2_, 95% O_2_) cutting solution [mM; 93 *N*-Methyl-D-glucamine (NMDG), 2.5 KCl, 1.2 NaH_2_PO_4,_ 30 NaHCO_3_, 20 HEPES, 25 glucose, 5 L-ascorbic acid, 2 thiourea, 3 Na-pyruvate, 10 MgSO_4_, 0.5 CaCl_2_, pH 7.35–7.4] (Ting et al., 2014) for 5 min. The heart was extracted and micro dissected to result in a thin layer of adipose and connective tissue containing the GP neurons and interconnecting nerves that lie superior to the posterior left atrium. The preparation was lightly digested with collagenase B (0.75 mg/mL, Sigma 110888) and trypsin (2.5 mg/mL, Sigma T4799) for 1 h at 37^°^C in carbogenated cutting solution to loosen the connective and adipose tissue surrounding the GP, and improve dye and drug access to the GP soma.

### Dye preparation and loading

The Ca^2+^ fluorescent dye Cal-520 AM (AAT Bioquest, 21130) was dissolved in 6.75 μL 20% (w/v) Pluronic F-127 in DMSO (Thermo Fisher, P3000MP, Carlsbad, CA, USA), made to 40 μL with recording solution (mM; 118 NaCl, 4.7 KCl, 1.13 NaH_2_PO_4_, 25 NaHCO_3_, 11.1 glucose, 1.3 MgCl_2_, 1.8 CaCl_2_) (Rimmer and Harper, 2006) and vortexed (15 min, RT). Recording solution was added to a final volume of 4 mL resulting in a final dye concentration of 0.01 mM (Gooch et al., 2019) with 0.003% (w/v) Pluronic F-127.

The atrial GP preparation was incubated with the Cal-520 AM dye solution for 2 h at 34^°^C in a chamber bubbled with carbogen (Brain Slice Keeper 2, Automate Science S-BSK2). The atrial GP preparation was then transferred to the recording chamber of a Zeiss Axioskop upright microscope that was perfused *via* gravity flow (2–3 mL/min) with carbogenated recording solution at 34^°^C. The sample was washed in the chamber for 10 min to remove excess dye before recording commenced.

### Drug application

Drug application was performed *via* a picospritzer using a microelectrode (4–7 MΩ resistance) positioned inside the GP in which recordings were being performed. Acetylthiocholine (ATCh, in recording solution at 500 μM, the concentration in the middle of the response amplitude dynamic range) (Cuevas and Berg, 1998), an acetylcholine mimetic, was applied at one bar pressure for 200 ms. The nicotinic receptor antagonist hexamethonium bromide [300 μM, a concentration previously shown to block nAChRs (Scott and Bennett, 1993; Ashton et al., 2020)] was bath applied *via* gravity feed and then washed away by application of recording solution for 20 min.

### Immunohistochemistry

Glial cells were detected by immunohistochemistry using methods detailed in our previous studies (Ashton et al., 2020). Briefly rat atrial GP preparations were fixed in 4% paraformaldehyde (PFA) overnight at 4^°^C and then cryoprotected with 20% sucrose (in 0.01 M phosphate buffered saline, PBS, 0.025% Azide) embedded in and frozen (−80^°^C), sectioned (20 μm) and mounted (Ashton et al., 2020). Sections were blocked (5% NDS, Sigma-Aldrich, D9663) in PBS 0.4% Triton X-100 (2 h, RT) and incubated with primary antibodies (1:50 choline acetyltransferase (ChAT) AB144P Goat Millipore; 1:100 S100B PA5-78161 Rat Thermo Fisher) in 1% NDS in PBS-T (overnight, 4^°^C), washed then incubated with secondary antibodies (Jackson) diluted in 1% NDS in PBS-T (1 h, RT) and washed. Sections were mounted and (Citifluor AF1, 17970-25) and stored at 4^°^C until needed.

Images were acquired with an Olympus FV1000 confocal using an oil immersion 60 × objective lens [numerical aperture (NA) = 1.35]. Gain and offset were optimized for each channel, and images were taken at 2 × zoom. 1,024 × 1,024 × 20 xyz pixel resolution was used.

### Calcium image acquisition and analysis

Imaging regions of interest were selected that contained ganglion clusters of five or more clearly defined GP neurons. Glial cells were identified as small diameter cells located immediately next to the GP and wrapping around the soma. Cal-520 AM fluorescence was excited with a mercury lamp (Zeiss mbq 52 AC 50 Watt), and images (672 × 512 pixels, detector size = 8.67 mm × 6.60 mm) were captured with a Digital CCD Camera (Hamamatsu ORCA-R2/C10600) system using an exposure of 100 ms for a final acquisition rate of 10 Hz with a 40x water immersion objective (Zeiss Achroplan Ph2, N.A. = 0.8). Excitation light intensity was adjusted to ensure that the full dynamic range of all responses was captured. Recording time was limited to a maximum of 12 min per ganglia.

Image analysis was performed using scripts written in MATLAB available at: https://github.com/Joscelin1/Calcium-Imaging-Analysis.git. Briefly, the recordings were motion-corrected, and the soma of each of the GP neurons was identified *via* a DIC image. GP soma were segmented in ImageJ (FIJI 4.0.5) and regions of interest were imported into MATLAB (R2019b) as masks to create an averaged soma intensity across time profile. A two step background subtraction was performed to eliminate baseline decay due to photobleaching: a 200 frame moving average trace of each region of interest (ROI) was subtracted from the original profile. Signal peaks were identified as amplitudes >3-fold the standard deviation of the background signal. A 1D mask was then created to identify areas within the original trace with strong fluorescence signal above background. The background region was then used to fit the background profile through to the original Z-axis profile with cubic spine interpolation (MATLAB). The background trace accurately followed the baseline decay caused by photobleaching and was subtracted from the original Z-axis profile.

Calcium dynamics were then measured (MATLAB); response amplitude (ΔF/F^0^) is the fluoresce change from baseline to maximum peak amplitude; duration at half maximum (s) is the response width at half the maximum amplitude; rise time (s) is time taken for the response to rise from 10 to 90% of the maximum peak amplitude; decay time (s) is the time taken for the response to fall from 90 to 10% of the maximum peak amplitude;% of initial amplitude/duration remaining with hexamethonium (Hex) is the maximum response amplitude/duration during hexamethonium application as a percentage of the initial response amplitude/duration before hexamethonium application.

### Statistical analysis

GraphPad Prism (Version 8.2.1) was used to calculate statistical significance. A Shapiro-Wilk Normality test was used to determine if the data had a gaussian distribution, and a Mann-Whitney test was selected for unpaired non-normally distributed data. Cumulative frequency was calculated to visualize potential differences in the spread of events Ca^2+^ events and calculated as the relative percentage of the frequency of each event amplitude, 25 and 75% percentiles are stated to quantify any change in histogram shape, and a Kolmogorov-Smirnov test was used to compare genotype differences in cumulative frequency distribution For multiple comparisons between genotypes and across hexamethonium treatment groups, where data was normally distributed a two-way ANOVA test was performed. Data are presented as mean ± standard error of the mean (SE) or median and 95% confidence intervals (CIs) where appropriate.

## Results

### Recording changes in somatic Ca^2+^ in rodent GP

**FIGURE 1:**
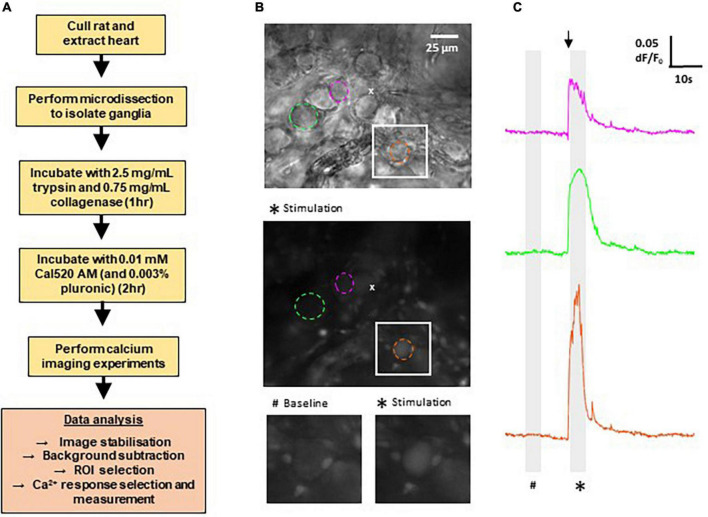
Methods for calcium imaging in rat ganglionated plexi (GP) neurons. **(A)** Flow chart outlining the major steps involved in recording Ca^2+^ responses from GP neurons in rat GP. **(B)** Top: differential interference contrast image of a WKY GP. The location of the micropipette used for acetylthiocholine (ATCh) application is indicated by (x). Middle: fluorescence image of the same region as above, labeled with Cal520 AM. Image shows a maximum intensity projection of 50 frames (5 s) during ATCh stimulation as indicated on the traces (*). Example regions of interest (ROIs) are selected around GP neuron soma (dashed circles). Colors correlate to corresponding Ca^2 +^ traces for each neuron in the right hand side column. Bottom: magnification of the white boxed area in the upper and middle images, highlighting a strongly responding neuron (orange). **(C)** Example traces of changes in Cal520 AM fluorescence measured in the ROIs in part **(B)** in response to ATCh stimulation (500 μM for 200 ms; black arrow). Traces show the baseline fluorescence (#, left) and the increase in fluorescence during ATCh stimulation (*, right).

Here we developed the techniques to record calcium transients in GP neurons in the intrinsic cardiac nervous system of WKY and SHRs. Figure 1 provides a summary of the methods involved, as well as example images of typical fields of view and example traces of the resulting measurements of fluorescence changes recorded in response to cholinergic stimulation. In total, the Ca^2+^ responses of WKY (4 WKY, 95 cells) and SHR (4 SHR, 80 cells) GP neurons were analyzed.

**FIGURE 2:**
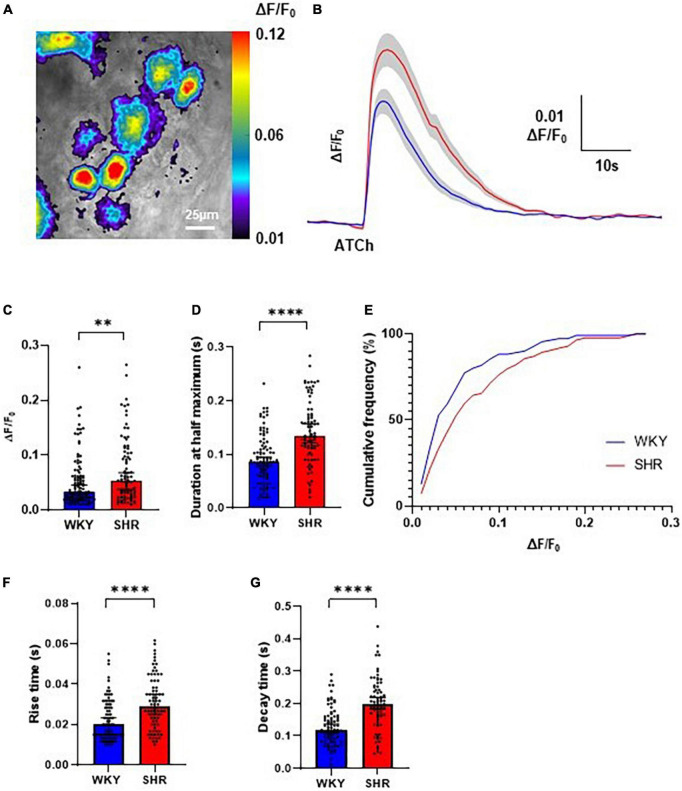
Evoked Ca^2+^ responses to acetylthiocholine (ATCh) application in Wistar Kyoto (WKY), and spontaneously hypertensive rat (SHR) ganglionated plexi (GP) neurons. **(A)** Example pseudo colour image of the fluorescent Ca^2+^ responses of SHR GP neurons to locally applied ATCh (200 ms, 500 μM). **(B)** Mean traces of SHR (red) and WKY (blue) neurons with ± SE highlighted in gray. **(C)** The peak amplitude (median ± 95% CI; Mann-Whitney test ^**^p < 0.01) and **(D)** duration at half maximum (median ± 95% CI; Mann-Whitney test ^****^p < 0.0001) of the evoked Ca^2+^ responses. **(E)** Cumulative frequency plot of response peak amplitude in WKY (blue) and SHR (Kolmogorov-Smirnov test) (red; 4 WKY, 95 cells; 4 SHR, 80 cells). **(F)** The rise time (s) (median ± 95% CI; Mann-Whitney test ^****^p < 0.0001) and **(G)** decay time (median ± 95% CI; Mann-Whitney test ^****^p < 0.0001) of the fluorescent calcium response of SHR (red) and WKY (blue) GP neurons to locally applied ATCh (200 ms 500 μM) (four WKY, 95 cells; four SHR, 80 cells).

Local application of ATCh (500 μM, 200 ms) was found to reliably evoke strong increases in Cal-520 fluorescence in GP soma in WKYs and SHRs, reflecting an increase in intracellular Ca^2+^ (Figures 1B, 2A). The observed fluorescence response to ATCh application was significantly larger in SHR compared to WKY GP neurons (Figures 2B–D), evident by a higher response amplitude [*p* = 0.0053; WKY 0.03304, 0.02885–0.04573 ΔF/F_0_; SHR 0.05351, 0.03801–0.06811 ΔF/F_0_ (median, 95% CI)] and longer duration [*p* < 0.0001; WKY 0.08611, 0.07944–0.09444 s; SHR 0.1344, 0.1217–0.1567 s (median, 95% CI)]. We also observed less smalland more large amplitude responses in SHRs compared to WKYs (25% percentile; WKY 0.02059, SHR 0.02726 vs. 75% percentile; WKY 0.06217 SHR 0.1041), resulting in a significant rightward shift in the cumulative frequency plot (*p* = 0.0254) (Figure 2E). The rise time [*p* < 0.0001; WKY 0.2000, 0.01500–0.02333 s; SHR 0.02917, 0.02500–0.03500 s (median, 95% CI)] and decay time [*p* < 0.0001; WKY 0.1183, 0.1083–0.1367 s; SHR 0.1967, 0.1833–0.2183 s (median, 95% CI)] of responses was also larger in SHRs compared to WKYs (Figures 2F, G). The greater overall Ca^2+^ response of SHRs relative to WKYs is consistent with the hypothesis that SHR GP neurons are more excitable (Ashton et al., 2020).

In contrast to the high amplitude evoked responses to ATCh, we found that spontaneous Ca^2+^ activity could not be detected within the GP neurons. Spontaneous Ca^2+^ fluorescent changes may be low amplitude events that were below our detection threshold. Alternatively, as previously observed in whole-cell patch clamp experiments (Ashton et al., 2020), GP neurons appear to have a low level of spontaneous activity in this preparation due to a lack of input from the CNS and cardiac afferents.

**FIGURE 3:**
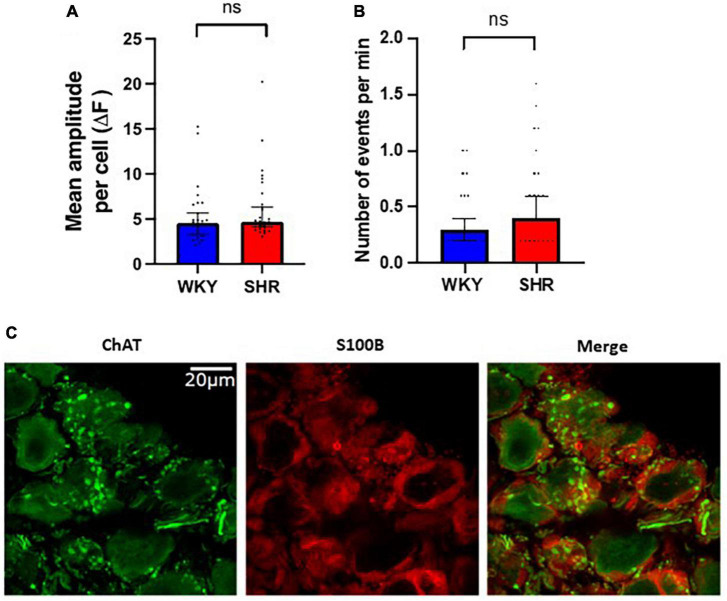
Spontaneous Ca^2+^ events in glial cells surrounding ganglionated plexi (GP) neurons. Fluorescence responses of putative glial cells in Wistar Kyoto (WKY) (blue) and spontaneously hypertensive rat (SHR) (red) GPs, showing **(A)** the mean amplitude response per cell (median ± 95% CI; Mann-Whitney test *p* > 0.05) and **(B)** the frequency of responses (median ± 95% CI; Mann-Whitney test *p* > 0.05) (5 WKY, 19 cells; five SHR, 23 cells). **(C)** Immunohistochemistry images of rat GP neurons showing ChAT positive (green) neuronal cell bodies and fibers encases by S100B positive (red) glial cells.

Interestingly spontaneous fluorescent Ca^2+^ events could be detected in the glial cells surrounding the GP neurons suggesting these cells are highly spontaneously active even in the absence of CNS input (Figure 3). The frequency of spontaneous glial Ca^2+^ events ranged from 0 to 1.6 Hz (Figure 3). No significant difference in spontaneous event frequency per minute was detected between WKYs and SHRs [*p* > 0.05; WKY 0.2998, 0.1999–0.3997; SHR 0.3997, 0.3996–0.5996 (median, 95% CI)]. In addition no significant difference was observed in spontaneous event amplitude per cell between WKYs and SHRs [*p* > 0.05; WKY 4.544, 3.284–5.655 ΔF/F_0_; SHR 4.700, 4.134–6.325 ΔF/F_0_ (median, 95% CI)].

### Cholinergic contribution to evoked Ca^2+^ responses in SHR and WKY GP neurons

**FIGURE 4:**
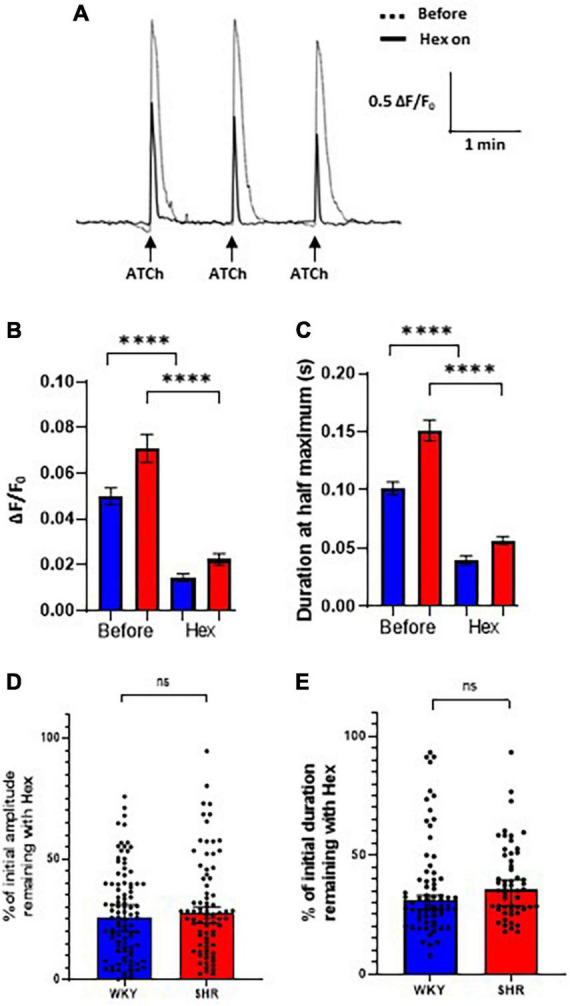
Hexamethonium block of acetylthiocholine (ATCh) evoked Ca^2+^ responses. Bath application of hexamethonium (300 μ M) reduced the ATCh (200 ms and 500 μm) evoked fluorescence responses in both Wistar Kyoto (WKY) and spontaneously hypertensive rat (SHR) ganglionated plexi (GP) neurons. **(A)** Example traces from a WKY GP neuron before (dashed) and during (solid black) hexamethonium application in response to three local, transient ATCh applications. **(B)** The amplitude (ΔF/F_0_) and **(C)** the duration at half maximum of responses in WKY (blue) and SHR (red) GP neurons were significantly reduced during hexamethonium application (mean ± SE; two-way ANOVA ^****^p < 0.0001). **(D)** The percentage of the initial response amplitude and **(E)** duration remaining after hexamethonium application in WKYs and SHRs (median ± 95% CI; Mann-Whitney test) (four WKY, 95 cells; three SHR, 67 cells). Only neurons that continued to respond with a response peak of >0.01 ΔF/F_0_ during hexamethonium treatment were included in the duration analysis **(C,E)**.

To assess the proportion of the Ca^2+^ response mediated by nAChRs, hexamethonium, a broad-spectrum nAChR antagonist, was applied to intact atrial GP preparations. The Cal-520 fluorescent response to ATCh application (200 ms, 500 μM) was partially blocked by bath application of hexamethonium (300 μM) in both WKY and SHR GP neurons (Figure 4). Spontaneous Ca^2+^ events in glial cells surrounding GP neurons. ATCh stimulation was performed three times before and during hexamethonium application (Figure 4A), and the average of the responses at each time point was taken to reduce the impact of interpeak variability. We note that addition of hexamethonium resulted in a significant overall reduction in the amplitude of the evoked ATCh response for both WKYs [Before 0.04994 ± 0.003796 ΔF/F_0_; hexamethonium 0.01451 ± 0.001624 ΔF/F_0_; (mean ± SE); *p* < 0.0001], and SHRs [Before 0.07093 ± 0.006092 ΔF/F_0_; hexamethonium 0.02240 ± 0.002589 ΔF/F_0_; (mean ± SE); *p* < 0.0001; Figure 4B].

The duration at half maximum of ATCh responses were also significantly reduced by hexamethonium (Figure 4C) in WKYs [Before 0.1013 ± 0.005583 s; hexamethonium 0.03966 ± 0.003934 s; (mean ± SE); *p* < 0.0001] and SHRs [Before 0.1512 ± 0.009133 s; hexamethonium 0.05675 ± 0.003584 s; (mean ± SE); *p* < 0.0001].

After hexamethonium application, the percentage of the remaining response amplitude in WKYs and SHRs was not significantly different (Figure 4D; *p* = 0.3913; WKY 25.90, 20.28–30.99%; SHR 27.49, 23.40–30.27% [median, 95% CI]) and duration [Figure 4E; *p* = 0.1480; WKY 31.15, 27.21–33.09%; SHR 35.54, 28.72–39.75% (median, 95% CI)]. These data show that nAChRs contribute the majority of the ATCh evoked Ca^2+^ responses in both SHRs and WKYs, but there is a significant nAChR independent component of the Ca^2+^ response remaining that is similar in SHRs and WKYs, which is aligned with previous experiments in dissociated rat ICNS neurons (Beker et al., 2003).

## Discussion

In this study we have provided an initial insight into the Ca^2+^ dynamics of GP neurons. We have shown that strong Ca^2+^ responses, mediated largely by nAChRs, can be evoked by cholinergic stimulation. Ca^2+^ responses were significantly larger in SHRs than in WKY controls, consistent with greater excitability of SHR GP neurons (Ashton et al., 2020).

### A new perspective on the ICNS

To the best of our knowledge, the Ca^2+^ changes of the intact atrial GP have not previously been recorded. Hence this method provides excellent potential for new research and insights into the functioning of the GP. This development now enables the recording of live Ca^2+^ responses in GP neurons in a rat *ex vivo* GP preparation. Existing studies looking at the properties of GP neurons have focused on immunohistochemistry and electrophysiology (Selyanko, 1992; Edwards et al., 1995; Ashton et al., 2020; Harper and Adams, 2021). While these techniques are useful for many applications, calcium imaging has the significant advantage of allowing simultaneous recordings of activity from multiple neurons in a given field of view. Calcium imaging experiments have been performed in dissociated cultured ICNS neurons by Beker et al. (2003), but this technique is limited as the neurons have lost their functional interconnections and synapses within the GP, significantly impacting their spontaneous and induced activity patterns. The GP integrates many different signals to regulate heart rate and rhythm; efferent, afferent, sympathetic and parasympathetic (Selyanko, 1992; Edwards et al., 1995; McAllen et al., 2011), so the neural network interactions within the GP are particularly important. However, it is essential to note that external input from the vagal and sympathetic nerves, as well as cardiac afferents, are not present in our preparation, and it is known that these inputs drive significant activity in the GP (McAllen et al., 2011). It will therefore be of interest in future studies to advance our calcium imaging techniques to a more intact preparation, such as the working heart-brainstem preparation (McAllen et al., 2011).

Interestingly this calcium imaging method is not limited to the neurons of the GP as we recorded significant spontaneous activity in glial cells that wrap around interconnecting fibres or are tightly associated with neuronal cell bodies (Nedergaard et al., 2010; Tedoldi et al., 2021). Ca^2+^ is suggested to provide a mechanism for glial-glial communication and glial-neuron communication (Nedergaard et al., 2010), and our observation of high glial Ca^2+^ activity suggests that these communication mechanisms are highly spontaneously active and likely influence the nearby GP neurons. In contrast to our data in GP neurons, we observed no difference in the frequency or amplitude of glial cell spontaneous activity between WKY and SHRs, suggesting the spontaneous activity of these cells in not influenced by hypertension and increased susceptibility to atrial arrhythmia. However, much is still unknown about the purpose and function of glial cells in the GP (Tedoldi et al., 2021), despite their abundance, providing an interesting future avenue of research.

### Ca^2+^ dynamics in GP neurons in SHR and WKY

Our data reveal a higher amplitude, longer duration and larger rise and decay times of cholinergic evoked Ca^2+^ responses in SHR compared to WKY GP neurons. Three main mechanisms likely contribute to this higher calcium signal: an elevated influx of Ca^2+^ through increased membrane-bound nAChRs, greater opening probability of voltage-gated Ca^2+^ channels, and/or the additional release of Ca^2+^ from internal stores. Recent immunohistochemical data has shown that SHRs have greater numbers of synaptic α7 nAChRs and lower numbers of β2 nAChR positive synapses relative to WKYs (Ashton et al., 2020). This change in the proportion of α7-nAChRs which are a highly Ca^2+^ permeable subtype (Fucile, 2004) likely contributes to the greater Ca^2+^ responses of SHRs. Several voltage-gated Ca^2+^ channels involved in neuron excitability and neurotransmitter release have also been identified in rat GP neurons (Jeong and Wurster, 1997; Catterall, 2011; Tu et al., 2014) and greater expression in SHRs compared to WKYs could also explain our observations. An *in vitro* study in cultured neonatal rat ICNS neurons showed that 40% of the Ca^2+^ response to ACh remained in Ca^2+^ free solution, indicating that a significant proportion of the Ca^2+^ response of GP neurons is reliant on internal stores (Beker et al., 2003). Furthermore, the mAChR dependent Ca^2+^ response was unaffected by reducing the external Ca^2+^ availability (Beker et al., 2003), suggesting these receptors are central to the internal Ca^2+^ release in GP neurons. M_1_–M_4_ mAChRs are expressed in the ICNS (Allen and Burnstock, 1990; Hassall et al., 1993), and a more detailed study on their role in the intact GP would be valuable due to their established connection to Ca^2+^ release and long-term changes in neuron excitation (Ishii and Kurachi, 2006; Vargas et al., 2011).

Consistent with this, hexamethonium blocked most but not all of the Ca^2+^ signal, with approximately 30% of the response amplitude and duration remaining in both SHRs and WKYs after hexamethonium application. This supports the notion that while the majority of the Ca^2+^ signal is mediated by nAChRs, the remaining Ca^2+^ response likely results from calcium influx through voltage-gated channels and/or mAChR induced Ca^2+^ release from internal Ca^2+^ stores (Beker et al., 2003). A more detailed study on the role of mAChRs in the GP would be valuable, especially considering their association with long-term changes in neuron excitation (Ishii and Kurachi, 2006; Vargas et al., 2011). A proportion of the remaining response may also be due to an incomplete block of the α7 nAChRs (Papke et al., 2010). We also observed that in WKYs and SHRs, the hexamethonium block was effective to different extents in different neurons within the same ganglia. This likely reflects a range of nAChR expression levels between GP neurons (Ashton et al., 2020), which was observed in previous immunohistochemical studies, and is likely to reflect the multiple subtypes of neurons found in the GP (Selyanko, 1992; Edwards et al., 1995; Klemm et al., 1997).

Elevated levels of Ca^2+^ in SHRs could potentially have widespread effects due to Ca^2+^’s important role in many cellular pathways (Sharma and Vijayaraghavan, 2003; Shen and Yakel, 2009; Evans and Blackwell, 2015). For example, high Ca^2+^ can lengthen the desensitization period of nAChRs in the short term, while it can alter gene expression in the long term (Shen and Yakel, 2009). Ca^2+^ also influences the induction and expression of synaptic plasticity (Evans and Blackwell, 2015), potentially impacting the synaptic connectivity of SHR GP neurons. Increased GP activity has been associated with episodes of AF (Scherlag et al., 2005; Po et al., 2006). Therefore, the greater SHR GP excitability due to larger intracellular Ca^2+^ responses may also be linked to AF. It is important to note that in our experiments, we are exposing a large proportion of the ganglia, and the cell membrane of each neuron, to ATCh at a potentially higher concentration and time frame. Under physiological conditions, ACh is released at the postsynaptic terminal and primarily functions within the synapse (Del Signore et al., 2004). Therefore, we need to consider that the activated receptors include synaptic as well as potentially peri- or extra-synaptic receptors that could be contributing to the observed calcium signals (Jones and Wonnacott, 2004).

## Conclusion

Our development of methods to utilize calcium imaging in intact atrial GP has enabled visualization of the Ca^2+^ responses of rat GP neuronal networks. This enables the investigation of network-wide responses of GP neurons, and the plasticity in these responses, for the first time. Overall, our data provide initial evidence for enhanced cholinergic-induced calcium signaling in SHR GP neurons. More broadly, our present and previous data have shown altered neuron synaptic structure and density, as well as altered physiological function and communication (Ashton et al., 2020) alongside greater neuronal excitability, as evidenced by Ca^2+^ dynamics and electrophysiology, which correlates with increased AF susceptibility in SHR (Ashton et al., 2020). This suggests that differences in GP neurons play a role in AF and may cause aberrant neural activity in the diseased GP. As overactivation of GPs promotes AF (Scherlag et al., 2005; Po et al., 2006; Báez-Escudero et al., 2014; Herring et al., 2019), the changes we have visualized may contribute to the underpinnings of this disease and it is important that further work can directly identify causation and the relative contribution of altered calcium dynamics in GP neurons and myocytes in AF.

## Data availability statement

The original contributions presented in this study are included in the article/supplementary material, further inquiries can be directed to the corresponding authors.

## Ethics statement

This animal study was reviewed and approved by University of Auckland Animal Ethics Committee.

## Author contributions

JS performed all experiments in Figures 1, 2, and 4 and drafted the manuscript. JA performed experiments in Figure 3. JA and LA assisted with experiment methods and manuscript preparation. JC assisted with data analysis methods. JM designed the study, supervised the study, and wrote the manuscript. All authors contributed to the article and approved the submitted version.
